# Cross-Linked Nanohybrid Polymer Electrolytes With POSS Cross-Linker for Solid-State Lithium Ion Batteries

**DOI:** 10.3389/fchem.2018.00186

**Published:** 2018-05-25

**Authors:** Jinfang Zhang, Xiaofeng Li, Ying Li, Huiqi Wang, Cheng Ma, Yanzhong Wang, Shengliang Hu, Weifeng Wei

**Affiliations:** ^1^School of Materials Science and Engineering, North University of China, Taiyuan, China; ^2^State Key Laboratory of Powder Metallurgy, Central South University, Changsha, China

**Keywords:** solid-state lithium ion batteries, cross-linked, polymer electrolyte, poly (ethylene oxide), POSS

## Abstract

A new class of freestanding cross-linked hybrid polymer electrolytes (HPEs) with POSS as the cross-linker was prepared by a one-step free radical polymerization reaction. Octavinyl octasilsesquioxane (OV-POSS) with eight functional corner groups was used to provide cross-linking sites for the connection of polymer segments and the required mechanical strength to separate the cathode and anode. The unique cross-linked structure offers additional free volume for the motion of EO chains and provides fast and continuously interconnected ion-conducting channels along the nanoparticles/polymer matrix interface. The HPE exhibits the highest ionic conductivity of 1.39 × 10^−3^ S cm^−1^, as well as excellent interfacial compatibility with the Li electrode at 80°C. In particular, LiFePO_4_/Li cells based on the HPE deliver good rate capability and long-term cycling performance with an initial discharge capacity of 152.1 mAh g^−1^ and a capacity retention ratio of 88% after 150 cycles with a current density of 0.5 C at 80°C, demonstrating great potential application in high-performance LIBs at elevated temperatures.

## Introduction

Currently, lithium ion batteries (LIBs) have attracted intensive attention by virtue of the high output voltage, low self-discharge rate, large energy density, no memory effect, and environmental friendly nature (Dunn et al., 2011; Qu et al., 2014; Xu, 2014; Zhang et al., 2018). As an important component of LIBs, the electrolyte affects the specific energy, charge/discharge performance, cycle life, safety performance, and production cost of the battery (Zhou et al., 2014; Li et al., 2017; Fan et al., 2018). The leakage and explosive nature of traditional organic electrolyte solvents for commercial LIBs could cause excessive charge/discharge, short circuit and overheat, resulting in the fire or explosion safety concerns (Quartarone and Mustarelli, 2011; Wang et al., 2017; Qu et al., 2018). Polymer electrolytes (PEs) show great advantages in broadening the working temperature range, extending the service life, improving safety performance and flexibility of multifunctional structure and shape design (Ramesh and Ling, 2010; Samulski, 2011; Young et al., 2014). However, compared with liquid electrolyte, PEs exhibit low ionic conductivity at room temperature (<10^−6^ S cm^−1^) and poor interfacial compatibility with electrode materials, resulting in the electrochemical cycle degradation and successive capacity fading (Nakayama et al., 2010; Khurana et al., 2014). Therefore, high performance electrolyte materials have become an active research area for lithium-ion battery applications.

Nanoparticle-containing hybrid polymer electrolytes (HPEs) by grafting/blending nanofillers, such as ceramic particles or surface-modified nanoparticles, have been considered as promising candidates to improve the ionic conductivity, since nanoparticles could provide new pathways for lithium ion migration (Ahn et al., 2003; Khurana et al., 2014; Shim et al., 2014). Furthermore, nanoparticles could act as a protective layer against interfacial side reactions to enhance the interfacial compatibility between electrode and electrolyte materials (Shim et al., 2015; Ma et al., 2016). Among them, polyhedral oligomeric silsesquioxane (POSS), consisting of inorganic framework and organic functional groups, has attracted significant interest as an effective nanofiller to improve the ion conductivity owing to its unique multiple chain-ended structure and highly ordered nanoscale organic/inorganic hybrid structure (Kuo and Chang, 2011; Wang et al., 2011). Several studies have shown that POSS-based HPEs exhibit high ionic conductivity and thermal stability and improved dimensional stability and interfacial compatibility, which demonstrates the potential application in LIBs (Kim et al., 2012, 2013; Kwon et al., 2014; Shim et al., 2014; Wei et al., 2014; Villaluenga et al., 2015). For example, Kwon et al. prepared organic/inorganic hybrid semi-interpenetrating network (semi-IPN) polymer electrolytes (HIPEs) based on poly(ethylene oxide-co-ethylene carbonate) (PEOEC) and POSS for all-solid-state lithium battery applications and the study showed that the HIPEs exhibit improvement in ionic conductivity along with enhanced dimensional stability due to the presence of the rigid and bulky POSS moiety (Kwon et al., 2014). Kim et al. have reported a series of organic/inorganic block and random hybrid polymer electrolytes containing POSS and PEG moieties, which exhibited enhanced ionic conductivity as well as dimensional stability due to the nanophase separation forming the ion-conducting channels (Kim et al., 2012).

**Scheme 1:**
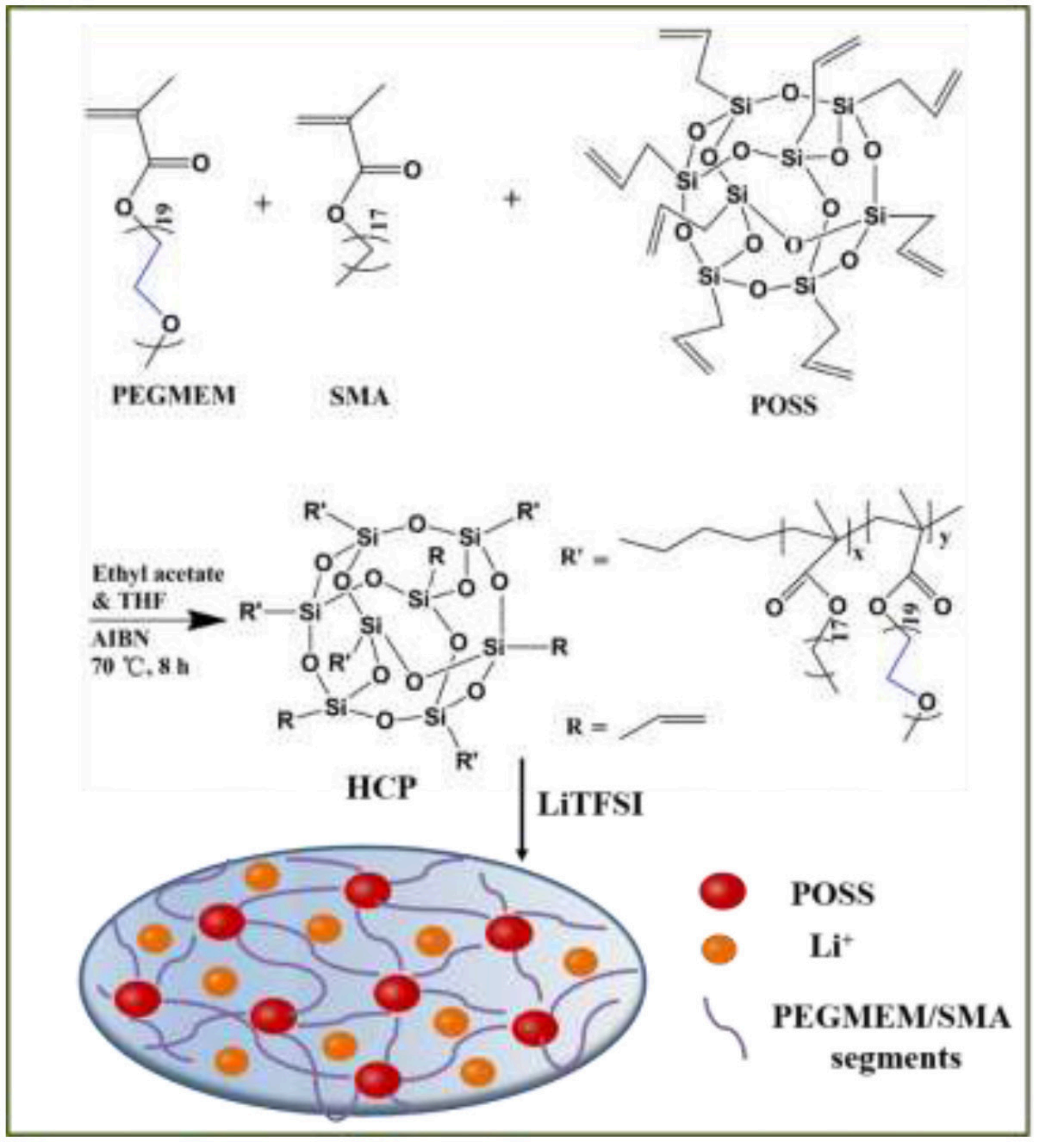
Synthesis of freestanding cross-linked HPE membrane.

Different from most of the reported polymer electrolytes with POSS as a nanofiller, here a new class of freestanding cross-linked HPE membranes with POSS as the cross-linker was prepared by a one-step free radical polymerization reaction based on our previous work (Zhang et al., 2016b). Octavinyl octasilsesquioxane (OV-POSS) with eight functional corner groups was used to provide cross-linker sites for the connection of polymer segment and the needed mechanical strength to separate the cathode and anode. Compared to PEGMEM/SMA polymer electrolyte, such cross-linked HPEs of PEGMEM/SMA-g-POSS with POSS as the cross-linker exhibit high ionic conductivity, stable interfacial compatibility and improved cycling stability and rate performance in solid-state LIBs.

## Experimental section

### Materials

Poly(ethylene glycol) methyl ether methacrylate (PEGMEM, *M*_*n*_ = 936 g mol^−1^), stearyl methacrylate (SMA, *M*_*n*_ = 338 g mol^−1^), octavinyl octasilsesquioxane (OV-POSS, *M*_*n*_ = 633 g mol^−1^) and lithium bis(trifluoromethane sulfonamide) (LiTFSI) were purchased from Sigma-Aldrich. LiTFSI was stored in a desiccator and dried under vacuum condition at 120°C for 12 h before used. Tetrahydrofuran (THF), 2,2-azobisisobutyronitrile (AIBN), ethyl acetate and petroleum ether, purchased from Sinopharm Chemical Reagent Co., Ltd., were used as received.

### Synthesis of PEGMEM/SMA-g-POSS copolymer

Organic/inorganic hybrid copolymers of PEGMEM/SMA-g-POSS were synthesized via free radical polymerization, as shown in Scheme 1. PEGMEM (2.5704 g, 85 wt%), SMA (0.4536 mL, 15 wt%) and AIBN (0.5 wt% of total mass) were dissolved in 8 mL of ethyl acetate to obtain solution A. Various amounts of OV-POSS (0.0775 g, 0.1592 g, 0.2452 g, 0.336 g) were dissolved in 8 mL of THF to form solution B. Solutions A and B were mixed in a 50 mL three-neck flask equipped with a condenser and the mixture was heated to 70°C in an oil bath under constant stirring and N_2_ atmosphere. After 10 h, the resultant mixture was dissolved in THF and precipitated in petroleum ether for three times. The final product was dried at 60°C for 12 h in vacuum oven and then the copolymers of PEGMEM/SMA-g-POSS were obtained.

### Prepare of the hybrid polymer electrolytes (HPEs)

PEGMEM/SMA-g-POSS copolymers and LiTFSI with a desired [EO]/[Li] molar ratio were dissolved in THF and then poured into the Teflon mold with groove. The mixed solution was transferred to the fume hood and stood for 12 h at room temperature. The HPEs were obtained after the mixture was dried in vacuum oven at 100°C for 12 h. And then the HPEs with cross-linked branching structure with POSS as the cross-linker were obtained, as shown in Scheme 1, and stored in glove box for further used. The thickness of polymer electrolyte membrane is around 200 μm.

### Materials characterizations and electrochemical measurements

^1^H NMR analysis (Avance III 400 MHz Digital NMR spectrometer) was used to characterize the structure of HCPs. Gel permeation chromatography (GPC) test (Waters 515–2410 instrument) was used to measure the molecular weight of HCP. Thermogravimetric analysis (TG) (TA-SDTQ600, 10°C min^−1^ under N_2_ flow) was performed to assess the thermal stability. Field emission scanning electron microscopy (FE-SEM) analysis (Nova Nano SEM230) was carried out to investigate the microstructure of electrolytes.

Symmetric stainless steel/HPE/stainless steel cells were assembled to measure the ionic conductivity of HPEs using a PARSTAT 4000 system (Ametek Advanced Measurement Technology INC.) over the frequency range of 0.1 Hz−100 kHz from 25 to 80°C with a perturbation voltage of 10 mV. Linear sweep voltammetry (LSV) was taken on stainless steel/HPE/Li cells to estimate the electrochemical stability of HPEs at a scan rate of 5 mV s^−1^. The symmetric Li/HPE/Li cells were fabricated to evaluate the interfacial stability between the electrolyte and lithium electrode. LiFePO_4_/HPE/Li coin cells were also assembled to investigate the electrochemical performance of HPEs in LIBs. Energy-type LiFePO4 was purchased from a reliable commercial source. The mixture of LiFePO_4_ (80 wt%), carbon black (10 wt%), and PVDF (10 wt%) was dispersed in N-methyl-2pyrrolidone (NMP) to form a slurry. Subsequently, the resultant slurry was coated onto aluminum foil, and then dried under vacuum at 110°C for 12 h to remove the residual NMP. The diameter of LiFePO_4_ cathode, electrolyte membrane and Li metal electrode in the coin cell is 12, 18, and 16 mm, respectively. The galvanostatic charge/discharge tests were carried out in a battery testing system (LANHE CT2001A, Wuhan LAND electronics Co., PR China) between 2.5 and 3.7 V at different rates.

## Results and discussion

### Synthesis and structural characterization of hybrid copolymers (HCPs)

**Figure 1:**
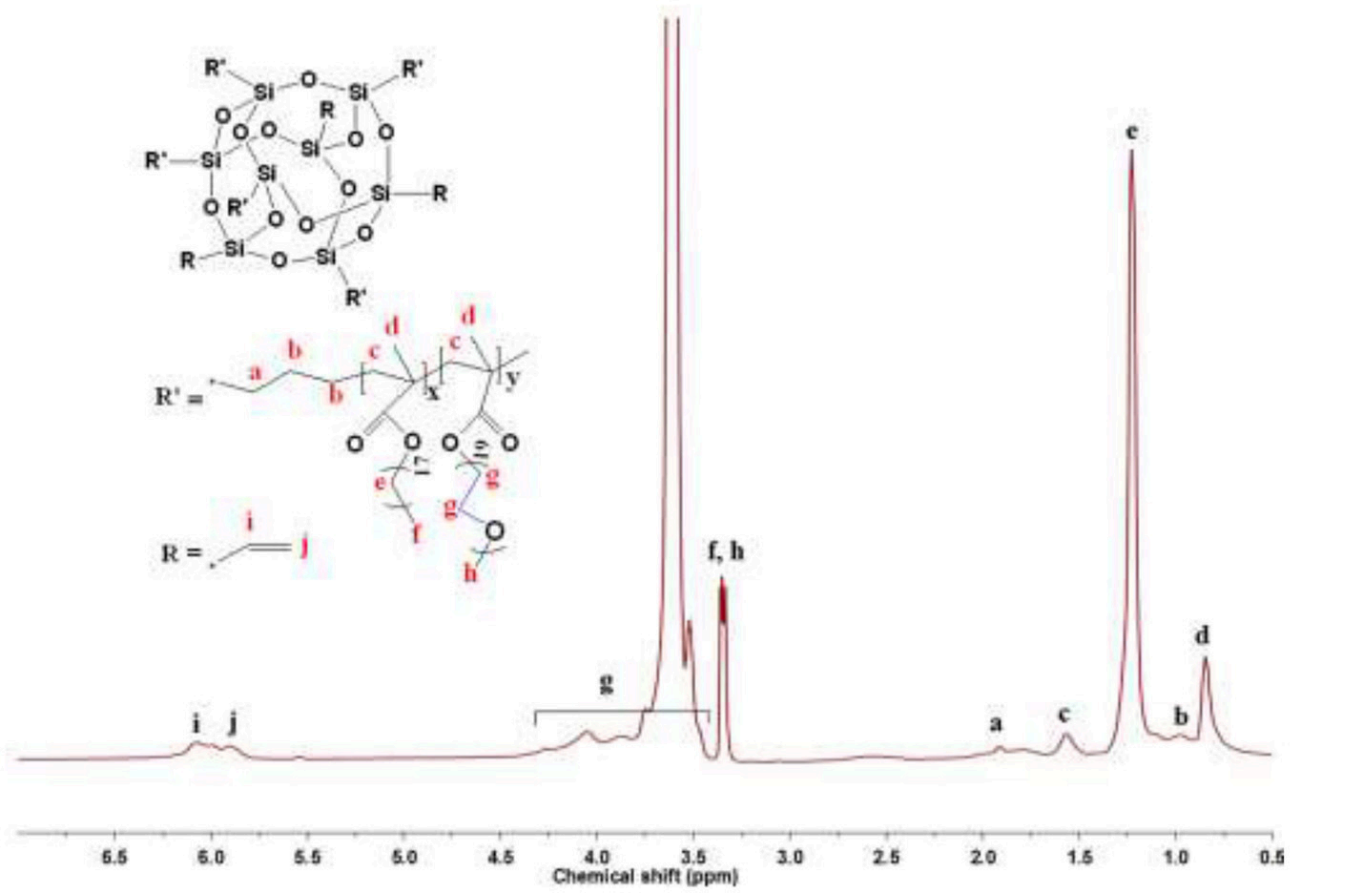
^1^H NMR spectrum of HCP-5.

^1^H NMR spectrum of the copolymers with the POSS molar ratio of 5% (HCP-5) is shown in Figure 1. All the resonance peaks in Figure 1 are assigned to each proton of the HCP-5. The signals of g at 3.41–4.32 ppm are assigned to the characteristic proton of CH_2_-CH_2_-O (EO) units in PEGMEM segments (Shim et al., 2014), while the resonance peak of e at 1.23 ppm is assigned to the characteristic proton of CH_2_ units in SMA segments (Zhang et al., 2016b). The protons from the reacted vinyl protons of POSS could be observed at 1.92 ppm (a). However, all the eight vinyls of POSS do not fully participate in the polymerization reaction since the multiple resonance signals of i and j at 5.88–6.07 ppm are assigned to the unreacted vinyl protons of POSS (Xu et al., 2005). The ^1^H NMR spectrum indicates that the HCPs combine the structural characteristic of PEGMEM, SMA and POSS. The POSS mole contents of HCPs can be estimated on the basis of ^1^H NMR spectrum, as shown in following equation (Equation 1):

(1) $$\begin{array}{l} \text{POSS mol}\%=[(I_{i+j}/3+{\text{I}}_{a}/2)/8]/[(I_{i+j}/3+{\text{I}}_{a}/2)/8\\ \;+I_{e}/34_{+}I_{g}/76] \end{array}$$

where *I*_*i*+*j*_ represents the integral area of the resonance peaks at 5.88–6.07 ppm from the unreacted vinyl protons of POSS, *I*_*a*_ represents the integral area of the resonance peaks at 1.92 ppm from the reacted vinyl protons of POSS, *I*_*e*_ represents the integral area of the resonance peak of protons from CH_2_ units in SMA segments at 1.23 ppm and *I*_*g*_ represents the integral area of the resonance peak from the protons of CH_2_-CH_2_-O (EO) units in PEGMEM segments at 3.41–4.32 ppm. The calculated values of various POSS mole contents are summarized in Table 1.

**Table 1:** Synthesis results of HCPs with different POSS contents.

**Samples**	**POSS (wt%)^a^**	**POSS (mol%)^a^**	**POSS (mol%)^b^**	**Yied (wt%)**	***Mw* (× 10^3^*gmol*^–1^)^c^**	**PDI^c^**
HCP-2.5	2.5	3.31	3.02	72.9	13.5	1.30
HCP-5	5	6.57	6.24	69.5	13.2	1.28
HCP-7.5	7.5	9.78	9.05	66.3	14.1	1.29
HCP-10	10	12.93	12.13	67.5	14.8	1.23

a *Feed ratio*.

b *Estimated based on ^1^H NMR spectroscopy*.

c Determined by GPC results.

Gel permeation chromatography (GPC) was used to measure the molecular weight of HCPs and the results are summarized in Table 1. The molecular weights (*M*_*w*_) of are in the range of 13.2–14.8 × 10^−3^ g mol^−1^ and values of polydispersity index (PDI) are ranging from 1.23 to 1.30, suggesting that the distribution of molecular weights for these HCPs was quite narrow.

### Morphology of HPEs

**Figure 2:**
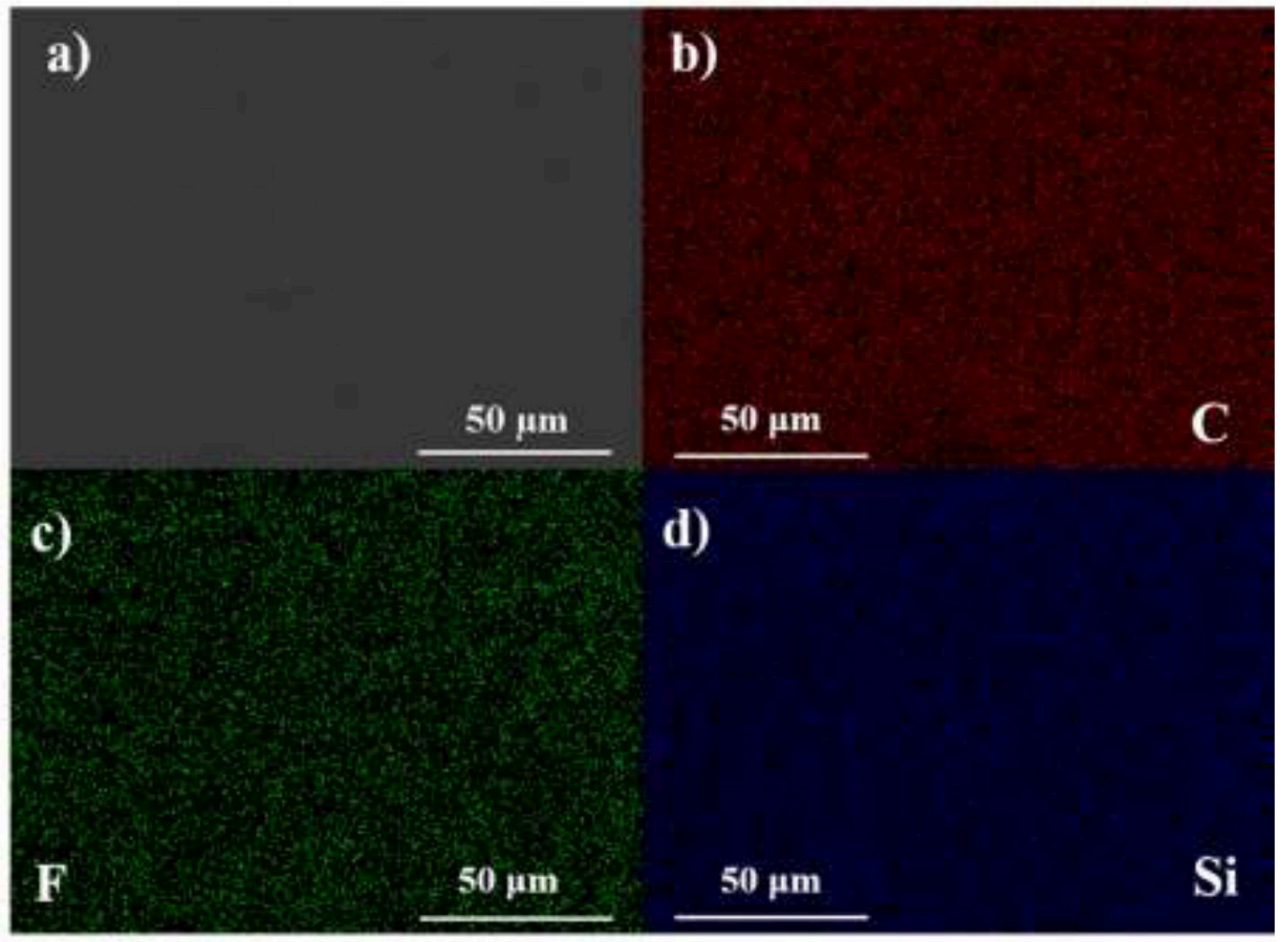
**(a)** SEM micrograph of HPE. **(b)** EDX mapping of C element. **(c)** EDX mapping of F element. **(d)** EDX mapping of Si element.

Figure 2 shows the SEM image and EDX mapping results of HPEs. Red represents C element mainly from PEGMEM chains, green represents F element derived from LiTFSI, and blue represents Si segments derived from OV-POSS. It can be found that the HPEs exhibit a smooth surface and the C, F, and Si elements dispersed in HPEs evenly, demonstrating that the HPEs were prepared successfully.

### Thermal and electrochemical stabilities

**Figure 3:**
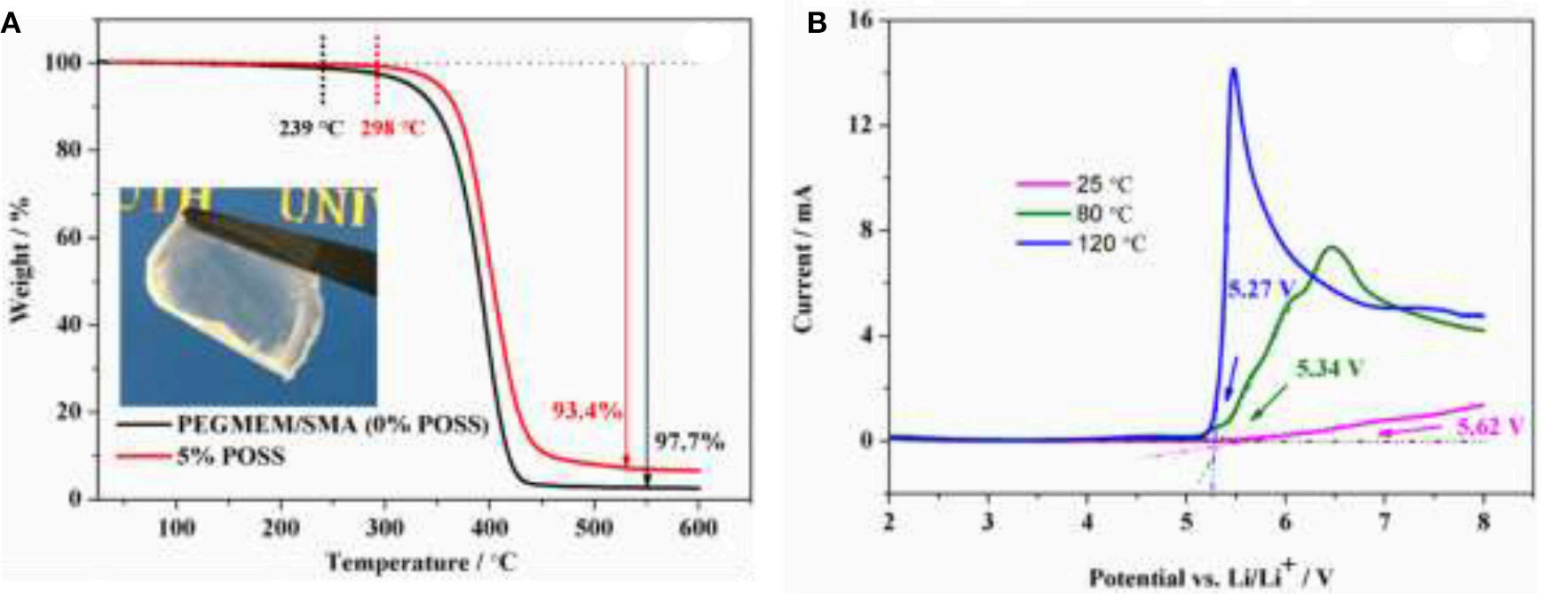
**(A)** Thermogravimetric curve of the hybrid polymer electrolyte containing 5% POSS andwithout POSS (0% POSS), and inset is the photograph of the hybrid polymer electrolyte containing 5% POSS; **(B)** LSV curve of the electrolyte based on HCP-5 at 25, 80, and 120°C.

Thermal and electrochemical stabilities are two important parameters used to estimate the safety of the electrolyte. As shown in the inset of Figure 3A, a colorless, transparent and freestanding electrolyte membrane was obtained. Figure 3A compares the TG results of the copolymer containing 5% POSS (HCP-5) and without POSS (PEGMEM/SMA) from 30 to 600°C. The degradation temperature of HCP-5 is 298°C while that of PEGMEM/SMA copolymer is 239°C, indicating that the addition of POSS moieties could improve the thermal stability of the copolymers (Xu et al., 2005). HCP-5 has a char yield of 6.6% at 600°C, which is higher than that of PEGMEM/SMA copolymer (2.3%) due to the combustion residues of POSS. The electrochemical window of the electrolyte based on HCP-5 is evaluated using LSV at 25, 80, and 120°C, as shown in Figure 3B. The oxidative decomposition potentials of 5.62, 5.34, and 5.27 V vs. Li/Li^+^ can be observed at 25, 80, and 120°C, respectively, suggesting that the electrolyte is less stable at high temperatures. Even so, such electrolyte still exhibits a high stable electrochemical window (>5.0 V vs. Li/Li^+^) at high temperatures, demonstrating great potential in high-potential LIB applications.

### Conductivity of HPEs

**Figure 4:**
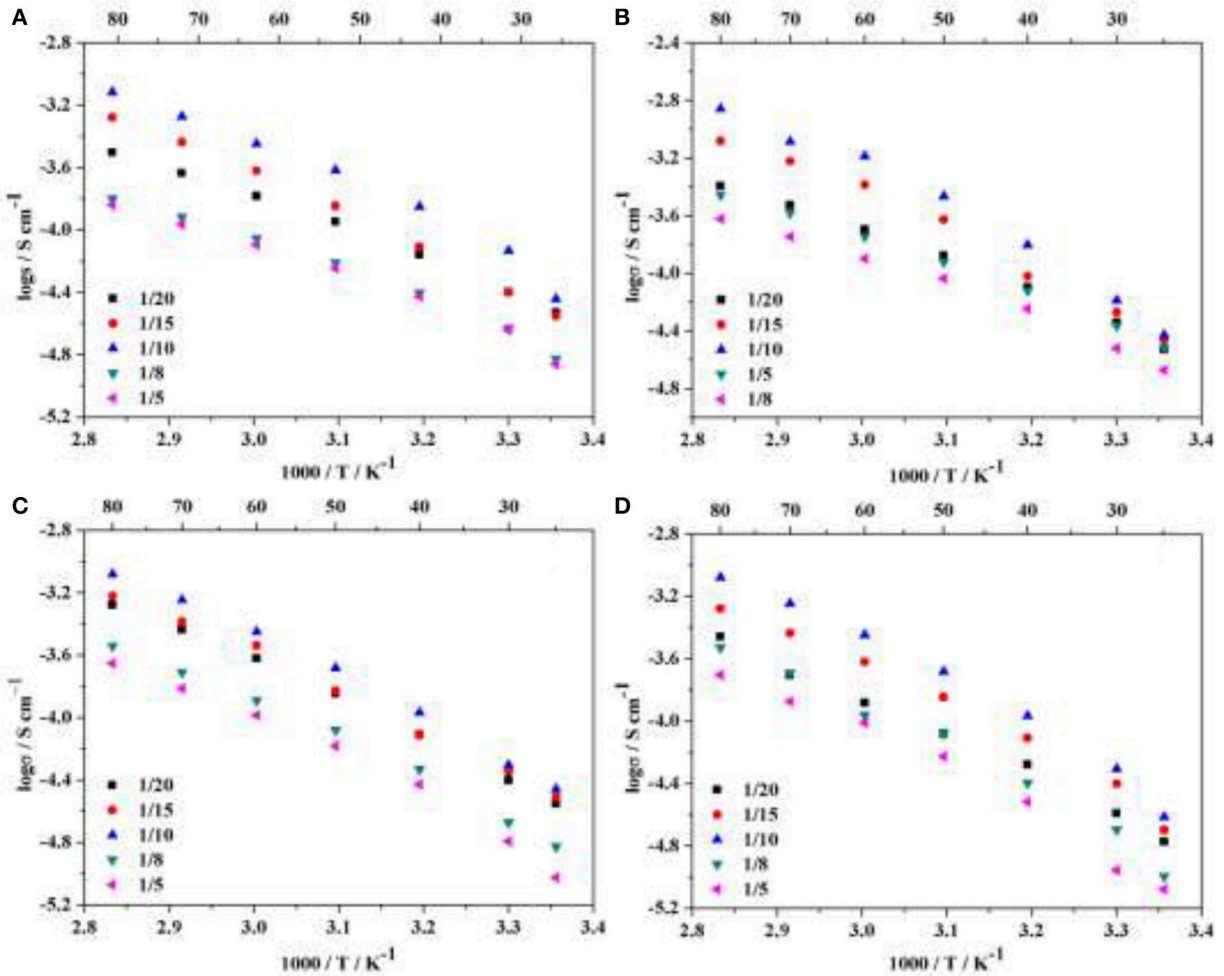
Temperature-dependent ionic conductivity of HPEs with various POSS contents at different LiTFSI concentrations: **(A)** 2.5 wt.% POSS, **(B)** 5 wt.% POSS, **(C)** 7.5 wt.% POSS, **(D)** 10 wt.% POSS.

Symmetric stainless steel/HPE/stainless steel cells were assembled to measure the ionic conductivity of HPEs using a PARSTAT 4000 system. The ionic conductivity was obtained by the following equation (Equation 2):

(2) $$\begin{array}{r} \sigma =\text{L}/\text{SR} \end{array}$$

where L and S were the thickness and area of the HPEs respectively, and R was the bulk resistance of the HPEs obtained from the Nyquist plot. The ionic conductivities of HPEs with various POSS contents (2.5 wt.% POSS, 5 wt.% POSS, 7.5 wt.% POSS, 10 wt.% POSS) at different LiTFSI concentrations were investigated, as shown in Figure 4. For all the samples, the ionic conductivity first increases with increasing the LiTFSI content, and then decreases with further increasing the lithium salt concentration. The maximum values of the conductivity were observed for HPEs with a [EO]/[Li] ratio of 10/1. Both the mobility of EO segments and the number of charge carriers have effect on the ionic conductivity of the electrolyte (Dissanayake et al., 2003; Panday et al., 2009). The number of charger carriers plays an active role at low LiTFSI content, so the ionic conductivity increases with increasing the LiTFSI content since the addition of LiTFSI could produce more charge carriers (Zhang et al., 2016a). However, when the concentration of LiTFSI exceeds a certain threshold, the number of effective charge carriers begins to decrease with increasing LiTFSI content since it may be prone to forming ion pairs or ion aggregates. Meanwhile, the increase of intermolecular interactions between the lithium cations and the EO chains could also lead to a decline in the mobility of EO chains (Singh and Bhat, 2003). As observed in other studies (Singh and Bhat, 2003; Ma et al., 2016; Zhang et al., 2016a,b), this phenomenon involves a trade-off between the number of charge carriers and the mobility of EO segments.

**Figure 5:**
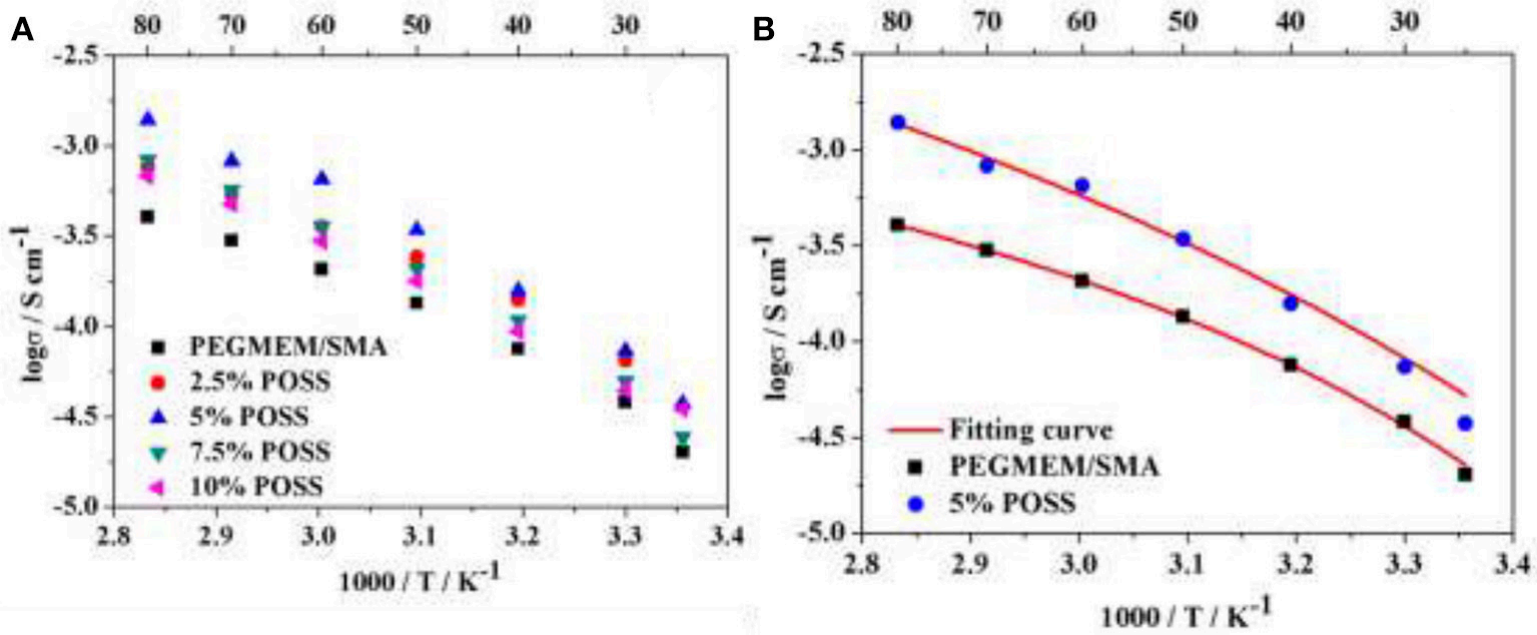
**(A)** Temperature-dependent ionic conductivity of HPEs with various POSS contents with [EO]/[Li] of 10/1; **(B)** VTF fitting results of HPEs based on HCP with 5% POSS and the PEGMEM/SMA copolymer.

**Figure 6:**
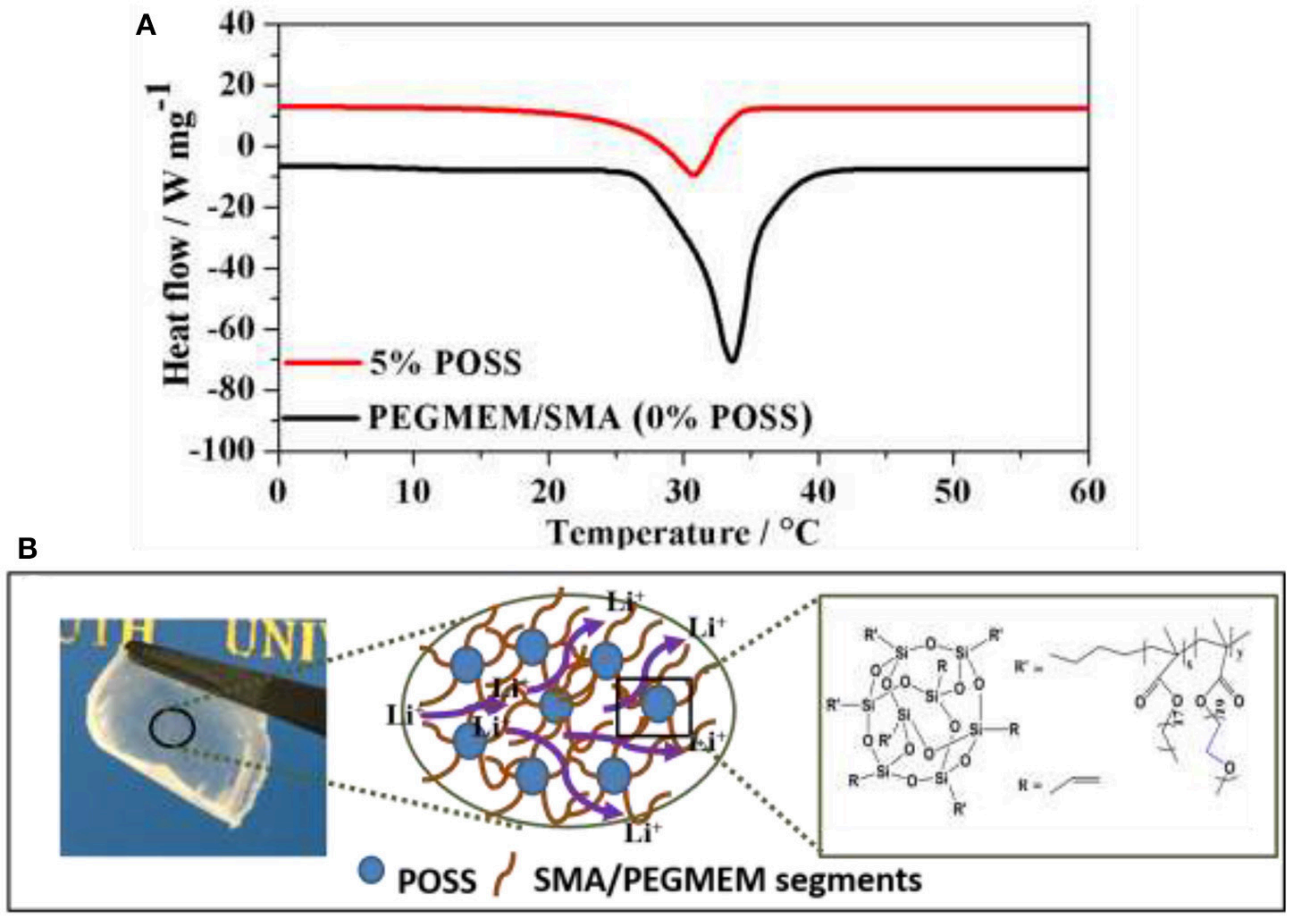
**(A)** DSC analysis of the hybrid polymer electrolyte containing 5% POSS and without POSS (0% POSS); **(B)** Schematic of lithium ion conduction mechanism designed for the cross-linked HPEs.

Figure 5A shows the temperature-dependent ionic conductivity of HPEs with various POSS contents at [EO]/[Li] of 10/1. It is noted that the ionic conductivity increases with the addition of POSS, up to a maximum when the POSS content is 5 wt.%, and then decreases with further increase of the POSS content. The HPE containing 5 wt.% POSS exhibits the highest ionic conductivity of 3.94 × 10^−5^ S cm^−1^ at 25°C and 1.39 × 10^−3^ S cm^−1^ at 80°C, much higher than that of the PEGMEM/SMA polymer electrolyte (Table 2) and other PEO based solid polymer electrolytes (Table S1). As discussed in other studies (Croce et al., 1998; Zhu et al., 2014), the addition of hybrid particles to polymer matrix with an optimized concentration results in the highest conductivity. The introduction of the POSS hybrid particles significantly improves the ionic conductivity by disrupting the order of EO segments and increasing the free volume for Li^+^ migration at low POSS content. Simultaneously, additional segmental motion of EO chains grafted on POSS particles provide new pathways along the nanoparticles/polymer matrix interface for lithium ion transport, as illustrated in Figure 6B. However, when the POSS particles content exceeds 5 wt.%, the ionic conductivity begins to decrease since the bulky POSS groups cannot provide channels for Li^+^ migration and the excess POSS nanoparticles will occupy the free volume, resulting in the decline of EO chain mobility.

**Table 2:** Ionic conductivity at 25 and 80°C and other parameters for the HPEs with various POSS contents at [EO]/[Li] of 10/1 derived from the VTF fit.

**Sample**	**Conductivity (S cm**^**−1**^**)**		**A**	***E*_a_**	***T_0_***
	**25^°^C**	**80^°^C**	**(S cm^−1^ K^1/2^)**	**(KJ mol^−1^)**	**(K)**
PEGMEM/SMA^a^	2.02 × 10^−5^	4.04 × 10^−4^	1.01	11.01	233
2.5 wt.% POSS	3.62 × 10^−5^	7.65 × 10^−4^	2.16	6.23	226
5 wt.% POSS	3.94 × 10^−5^	1.39 × 10^−3^	3.64	4.01	213
7.5 wt.% POSS	2.43 × 10^−5^	8.33 × 10^−4^	1.87	6.22	219
10 wt.% POSS	3.49 × 10^−5^	6.79 × 10^−4^	1.69	7.35	224

a *From our previous work (Zhang et al., 2016b)*.

Temperature-dependent ionic conductivity of HPEs obeys the Vogel–Tamman–Fulcher (VTF) equation (Equation 3):

(3) $$\begin{array}{r} \sigma \;=\text{A}T^{-1/2}\;exp\frac{-E_{a}}{k\left(T-T_{0}\right)} \end{array}$$

Where A is the pre-exponential factor, reflecting the number of charge carriers (Ratner et al., 2000); *T* is the testing temperature; *E*_*a*_ is the activation energy; *k* is Boltzmann constant (8.314 × 10^−3^ kJ mol^−1^ K^−1^) and stands for is the equilibrium glass-transition temperature (Panday et al., 2009).

Table 2 shows the ionic conductivity at 25 and 80°C and other parameters for the HPEs with various POSS contents at [EO]/[Li] of 10/1 derived from the VTF fit. The highest value of A (3.64 S cm^−1^ K^1/2^) and conductivity (3.94 × 10^−5^ S cm^−1^ at 25°C and 1.39 × 10^−3^ S cm^−1^ at 80°C) are observed for the HPE containing 5 wt.% POSS, indicating that the introduction of POSS could facilitate the transmission of charge carriers by increasing free volume for the motion of EO chains. However, the ionic conductivity decreases with the further increase of POSS, since the bulky POSS segments act as a barrier to restrain the migration of charge carriers. The value of *E*_*a*_ in Table 2 is between 4.01 and 11.01 kJ mol^−1^, which is in agreement with the value for solid polymer electrolyte (Lin et al., 2013; Wu et al., 2014; Daigle et al., 2015). The lowest *E*_*a*_ (4.01 KJ mol^−1^) and *T*_0_ (213 K) also could be observed for the HPE containing 5 % POSS. Figure 5B shows the comparison between the experimental data and VTF fitting results for HPE with 5 % POSS and PEGMEM/SMA copolymer and it could be observed that the experimental result of conductivity is definitely consistent with the fitting equation. Consequently, the HPE with 5% POSS content at [EO]/[Li] of 10/1 exhibits the highest conductivity and was used for the following electrochemical tests.

The improvement in ionic conductivity for the HPE with 5% POSS content can be attributed to the reduced crystallinity of the EO groups. Figure 6A shows the typical DSC analysis of the hybrid polymer electrolyte containing 5% POSS and without POSS (0% POSS). Various parameters of melting temperature (*T*_*m*_), melting enthalpy (ΔH_m_) and degree of crystallinity (*X*_*c*_) obtained from DSC are summarized in Table S2. Notably, the hybrid polymer electrolyte containing 5% POSS possesses the lower degree of crystallinity, which could be attributed to the strong interactions between polymer segments and POSS nanoparticles. In contrast to PEGMEM/SMA polymer electrolyte, PEGMEM/SMA-g-POSS copolymer electrolyte exhibits unique cross-linked structure and the steric hindrance effect among POSS groups offers additional free volume for the motion of EO chains. Previous research in our laboratory has showed that the addition of POSS increase the amorphous fraction of the copolymer (Zhang et al., 2016a), and the unique cross-linked structure also facilitates the reduction of the crystallinity of EO segments (Ma et al., 2016). Meanwhile, the branches of SMA and PEGEME segments grafted on the surface of the POSS particles allow for additional segmental motion and provide low energy, fast and continuously interconnected ion-conducting channels along the nanoparticles/polymer matrix interface for Li^+^ transport, resulting in higher ionic transmission efficiency, as schematically illustrated in Figure 6B.

### Interfacial stability of HPE/Li interface

**Figure 7:**
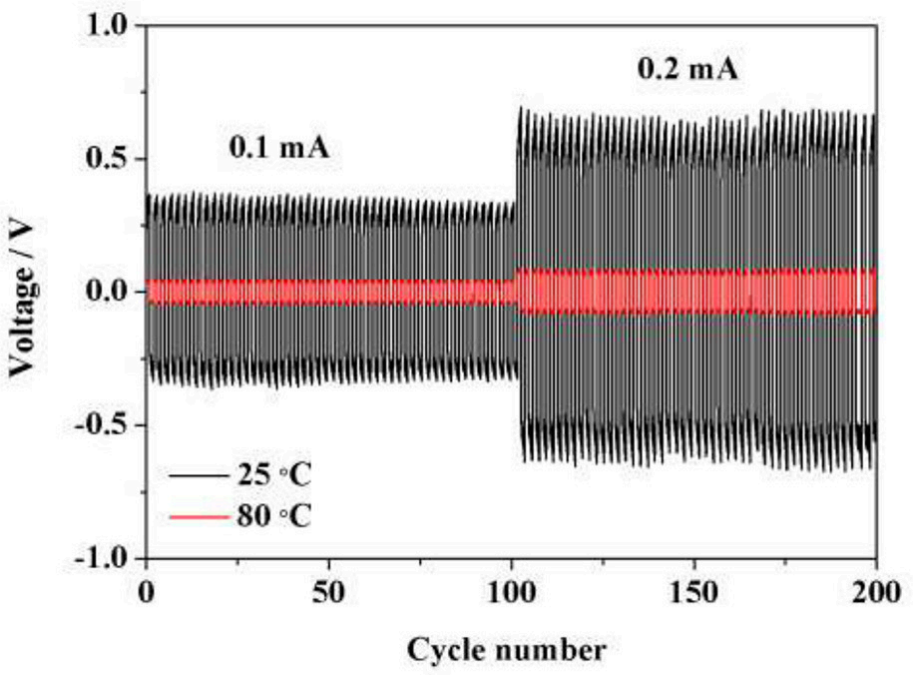
Polarization test for Li/Li symmetric battery with a constant current density of 0.1 and 0.2 mA cm^−2^ at 25 and 80°C.

Interfacial stability between polymer electrolyte and electrode materials is the key factor to affect the performance of lithium ion batteries. Polarization testing with a constant current density of 0.1 mA cm^−2^ for a Li/Li symmetric cell was carried out to investigate the interfacial stability at 25 and 80°C. As shown in Figure 7, the polarization voltage at 0.1 mA cm^−2^ is ~0.698 and 0.046 V at 25 and 80°C, respectively. When the current density increases to 0.2 mA cm^−2^, polarization voltage presents ~1.288 and 0.144 V at 25 and 80°C, respectively. No obvious fluctuations could be observed during the whole cycling process, demonstrating that such HPE exhibits stable interfacial compatibility with lithium metal electrode and shows great potential for application in high performance LIBs.

### Battery performance

**Figure 8:**
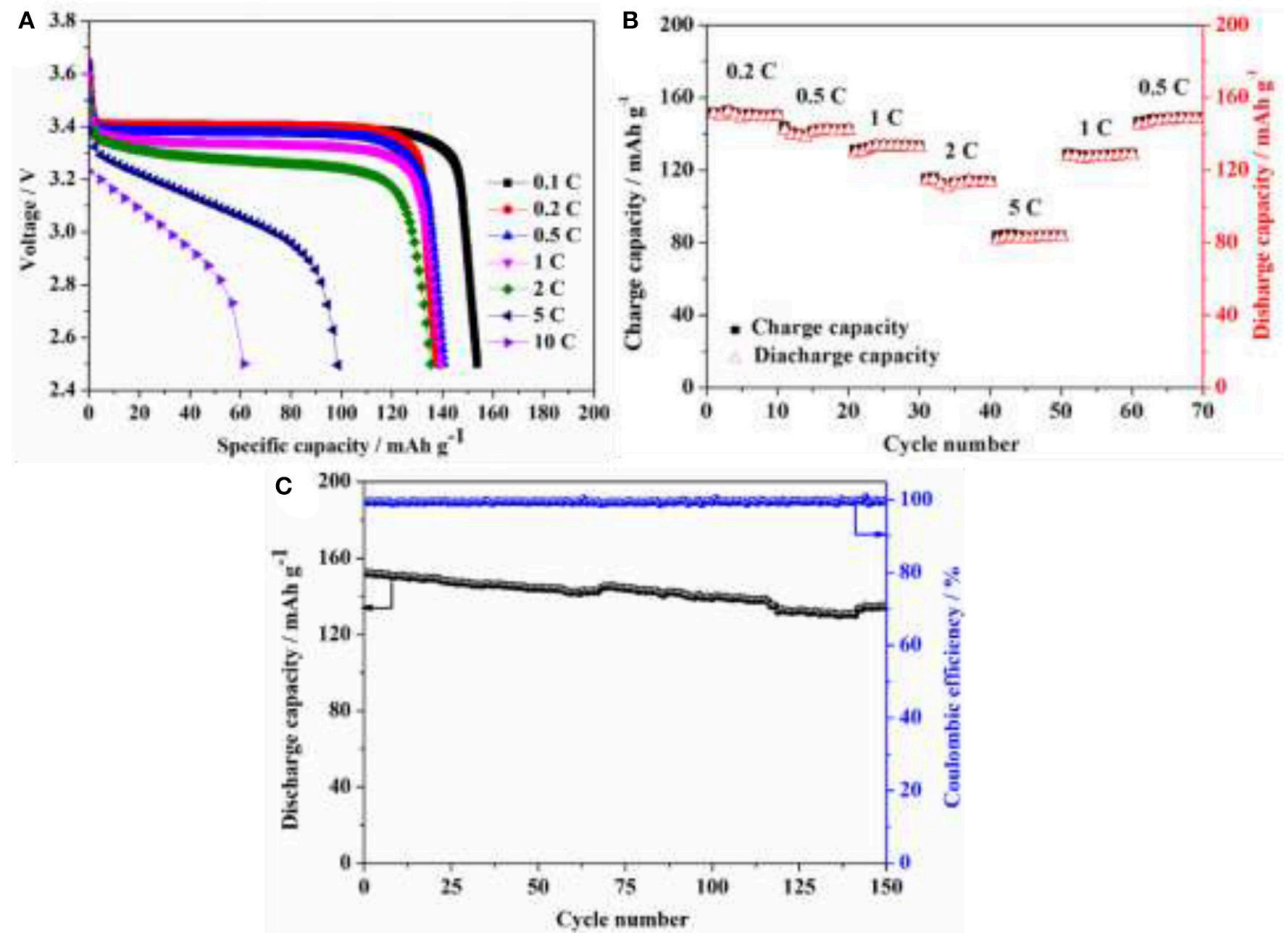
**(A)** Discharge curves for LiFePO_4_/Li battery assembled using HPEs with 5% POSS content under various C-rates with a charge current density of 0.1 C at 80°C; **(B)** Charge and discharge performance of LiFePO_4_/Li battery assembled using HPEs with 5% POSS content under different circulatory rates at 80°C; **(C)** Cycling performance of LiFePO_4_/Li battery assembled using HPEs with 5% POSS content with a current density of 0.5 C at 80°C.

LiFePO_4_/Li coin cells using HPEs with 5 wt.% POSS content were assembled for evaluating the battery performance. Figure 8A shows the discharge curves for LiFePO_4_/Li coin cell under various C-rates with a charge current density of 0.1 C at 80°C. Discharge capacities of 154 mAh g^−1^ were achieved at 0.1 C and ~140 mAh g^−1^ could be observed at 0.2, 0.5, 1, and 2 C, and the flat potential plateaus could also be observed up to 2 C rate. Even at higher discharg rates of 5 and 10 C, the discharge capacities of 99 and 61 mAh g^−1^ could still be obtained, respectively. Figure 8B presents a comparison of charge and discharge performance under different circulatory rates. The capacity decreases when the current density increases from 0.2 C to 5 C, and then recovers to its original value when the current density back to 0.2 C. Figure 8C presents the long-term cycling performance of LiFePO_4_/Li battery assembled using HPEs with 5 wt.% POSS content with a current density of 0.5 C at 80°C. Note that the cell delivers an initial discharge capacity of 152.1 mAh g^−1^ with a capacity retention ratio of 88% after 150 cycles and the coulombic efficiency of ~99% could be observed in the charge/discharge process, which is higher than other PEO-based solid polymer electrolyte (Table S1). The results indicate that the cells display great potential application in high-performance LIBs at elevated temperatures.

## Conclusion

A new class of freestanding cross-linked HPEs with POSS as the cross-linker was prepared by a one-step free radical polymerization reaction. PEGMEM/SMA-g-POSS HPEs exhibit unique cross-linked structure and provide low energy, fast and continuously interconnected ion-conducting channels along the nanoparticles/polymer matrix interface. Compared to PEGMEM/SMA polymer electrolyte, such cross-linked HPEs exhibit enhanced thermal and electrochemical stabilities, improved ionic conductivity and interfacial compatibility. Moreover, LiFePO_4_/Li cells assembled using HPEs with 5 wt.% POSS content delivers not only good rate capability but also enhanced long-term cycling performance at 80°C, displaying great potential application in high-performance LIBs at elevated temperatures.

## Author contributions

JZ and CM carried out the experimental work and the data collection and interpretation. XL participated in the design and coordination of experimental work, and acquisition of data. YL and HW participated in the study design, data collection, analysis of data and preparation of the manuscript. YW, SH, and WW carried out the study design, the analysis and interpretation of data and drafted the manuscript. All authors read and approved the final manuscript.

### Conflict of interest statement

The authors declare that the research was conducted in the absence of any commercial or financial relationships that could be construed as a potential conflict of interest. The reviewer, BQ, and handling Editor declared their shared affiliation.
