# Supplementary material for: Cross-Linked Nanohybrid Polymer Electrolytes With POSS Cross-Linker for Solid-State Lithium Ion Batteries
Source: Front Chem. 2018 May 25;6:186. doi: 10.3389/fchem.2018.00186 (PMC5981318; doi:10.3389/fchem.2018.00186)
Supplement: Supplementary file 1 [file Table_1.DOCX]

**Table S1** Comparative data of conductivity, initial discharge capacity at 0.5 C for LiFePO_4_/PEO based solid polymer electrolytes/Li cells at 25 and 80 ^o^C

| Ref. | Temperature  ^o^C | Conductivity  (S cm^-1^) | Initial discharge capacity at 0.5 C  (mAh g^-1^) |
| --- | --- | --- | --- |
| ([Yuan et al., 2013](#_ENREF_5)) | 25 | 3.16 ×10^-5^ | unknown |
| ([Wang et al., 2010](#_ENREF_4)) | 25 | ~2 × 10^-6^ | unknown |
| ([Zhu et al., 2014](#_ENREF_7)) | 80 | 9.71× 10^-4^ | unknown |
| ([Bouchet et al., 2013](#_ENREF_1)) | 80 | 2.5 ×10^-5^ | ~146 |
| ([Lu et al., 2013](#_ENREF_2)) | 80 | 1.14 ×10^-3^ | ~ 118 |
| ([Zhang et al., 2016](#_ENREF_6)) | 25 | 2.54 ×10^-5^ | unknown |
| ([Zhang et al., 2016](#_ENREF_6)) | 80 | 2.49 ×10^-4^ | unknown |
| ([Ma et al., 2016](#_ENREF_3)) | 25 | 7.4 ×10^-5^ | ~100 |
| **This work** | **25** | **3.94 × 10^-5^** | **unknown** |
| **This work** | **80** | **1.39 × 10^-3^** | **152.1** |

**Reference**

Bouchet, R., Maria, S., Meziane, R., Aboulaich, A., Lienafa, L., Bonnet, J.-P., et al. (2013). Single-ion BAB triblock copolymers as highly efficient electrolytes for lithium-metal batteries. *Nature materials* 12(5)**,** 452-457. doi: 10.1038/nmat3602.

Lu, Q., Fang, J., Yang, J., Yan, G., Liu, S., and Wang, J. (2013). A novel solid composite polymer electrolyte based on poly(ethylene oxide) segmented polysulfone copolymers for rechargeable lithium batteries. *Journal of Membrane Science* 425-426**,** 105-112. doi: 10.1016/j.memsci.2012.09.038.

Ma, C., Zhang, J., Xu, M., Xia, Q., Liu, J., Zhao, S., et al. (2016). Cross-linked branching nanohybrid polymer electrolyte with monodispersed TiO2 nanoparticles for high performance lithium-ion batteries. *Journal of Power Sources* 317**,** 103-111. doi: 10.1016/j.jpowsour.2016.03.097.

Wang, L., Li, X., and Yang, W. (2010). Enhancement of electrochemical properties of hot-pressed poly(ethylene oxide)-based nanocomposite polymer electrolyte films for all-solid-state lithium polymer batteries. *Electrochimica Acta* 55(6)**,** 1895-1899. doi: 10.1016/j.electacta.2009.11.003.

Yuan, C., Li, J., Han, P., Lai, Y., Zhang, Z., and Liu, J. (2013). Enhanced electrochemical performance of poly(ethylene oxide) based composite polymer electrolyte by incorporation of nano-sized metal-organic framework. *Journal of Power Sources* 240**,** 653-658. doi: 10.1016/j.jpowsour.2013.05.030.

Zhang, J., Ma, C., Xia, Q., Liu, J., Ding, Z., Xu, M., et al. (2016). Composite electrolyte membranes incorporating viscous copolymers with cellulose for high performance lithium-ion batteries. *Journal of Membrane Science* 497**,** 259-269. doi: 10.1016/j.memsci.2015.09.056.

Zhu, K., Liu, Y., and Liu, J. (2014). A fast charging/discharging all-solid-state lithium ion battery based on PEO-MIL-53 (Al)-LiTFSI thin film electrolyte. *RSC Advances* 4(80)**,** 42278-42284.
