# Supplementary material for: Cross-Linked Nanohybrid Polymer Electrolytes With POSS Cross-Linker for Solid-State Lithium Ion Batteries
Source: Front Chem. 2018 May 25;6:186. doi: 10.3389/fchem.2018.00186 (PMC5981318; doi:10.3389/fchem.2018.00186)
Supplement: Supplementary file 2 [file Table_2.DOCX]

**Table S2**. Thermal data for the hybrid polymer electrolyte containing 5% POSS and without POSS (0% POSS).

|  | *T_m_* / ℃ | *ΔH_m_* / J g^-1^ | χ_c_ |
| --- | --- | --- | --- |
| PEGMEM/SMA (0%POSS)  5% POSS | 33.42  30.82 | 100.9  75.13 | 0.51  0.38 |

*The reference melting enthalpy of 100% crystalline PEO is 195 J mol^-1^ ([Beaudoin et al., 2013](#_ENREF_1)^)^

**Reference**

Beaudoin, E., Phan, T.N., Robinet, M., Denoyel, R., Davidson, P., Bertin, D., et al. (2013). Effect of interfaces on the melting of PEO confined in triblock PS-b-PEO-b-PS copolymers. *Langmuir* 29(34)**,** 10874-10880. doi: 10.1021/la401889h.
